# Cytogenetic testing by fluorescence in situ hybridization is improved by plasma cell sorting in multiple myeloma

**DOI:** 10.1038/s41598-022-11676-w

**Published:** 2022-05-18

**Authors:** Jihye Ha, Hyunsoo Cho, Taek Gyu Lee, Saeam Shin, Haerim Chung, Ji Eun Jang, Soo-Jeong Kim, June-Won Cheong, Seung-Tae Lee, Jin Seok Kim, Jong Rak Choi

**Affiliations:** 1 Department of Laboratory Medicine, Hanyang University College of Medicine, Seoul, Republic of Korea; 2 Department of Internal Medicine, Yonsei University College of Medicine, Severance Hospital, 50 Yonsei-ro, Seodaemun-gu, Seoul, 03722 Republic of Korea; 3 Graduate School of Medical Science, Brain Korea 21 PLUS Project, Yonsei University College of Medicine, Seoul, Republic of Korea; 4 Department of Laboratory Medicine, Yonsei University College of Medicine, Severance Hospital, 50 Yonsei-ro, Seodaemun-gu, Seoul, 03722 Republic of Korea; 5 Dxome Co. Ltd. 8, Seongnam-daero 331beon-gil, Bundang-gu, Seongnam-si,, Gyeonggi-do Republic of Korea

**Keywords:** Myeloma, Cytogenetics

## Abstract

Accurate detection of cytogenetic abnormalities has become more important for improving risk-adapted treatment strategies in multiple myeloma (MM). However, precise cytogenetic testing by fluorescence in situ hybridization (FISH) is challenged by the dilution effect of bone marrow specimens and poor growth of plasma cells ex vivo. It has been suggested that FISH should be performed in combination with plasma cell enrichment strategies. We examined cytogenetic abnormalities in newly diagnosed MM and compared the efficacy of three different enrichment modalities for FISH: direct FISH (n = 137), fluorescence immunophenotyping and interphase cytogenetics as a tool for the investigation of neoplasms (FICTION) technique (n = 224), and a plasma cell sorting FISH with fluorescence-activated cell sorter (FACS) (n = 132). FISH disclosed cytogenetic abnormalities in 38.0% of samples by direct FISH, 56.3% by FICTION, and 95.5% by FACS-FISH, and the percentage of cells with abnormal signals detected by FISH was significantly higher by FACS-FISH than direct FISH or FICTION. Our results suggest that the efficacy of FISH is dependent on the plasma cell enrichment modalities and reveal that plasma cell sorting FISH with FACS enables better detection of cytogenetic abnormalities in diagnostic MM samples.

## Introduction

Multiple myeloma (MM) is a clonal B-cell lymphoproliferative neoplasm characterized by proliferation of malignant plasma cells (PCs) in the bone marrow (BM)^1^. Since MM is a heterogeneous disease with a variable clinical course, precise risk stratification is essential for prognostication, treatment decision, and improving patient outcome. The presence of t(4;14), t(14;16), or deletion 17p is classified as high-risk MM in the Revised International Staging System (R-ISS), and t(14;20) and gain 1q are also regarded as high-risk cytogenetic features in The Mayo Stratification of Myeloma and Risk-Adapted Therapy (mSMART), which are delineated by cytogenetic testing with fluorescence in situ hybridization (FISH)^2,3^.

Challenges in cytogenetic testing in MM mainly come from the dilution effect of BM aspirates and poor growth of plasma cells under ex vivo culture, making it difficult to selectively obtain metaphase chromosomes from malignant plasma cells^4^. However, FISH can overcome some of these limitations as the yield of interphase FISH is independent of plasma cell proliferative activity^4,5^. Furthermore, low plasma cell frequency in BM aspirates of MM patients can be enriched by fluorescence-activated cell sorting (FACS), magnetic-activated cell sorting (MACS), or selection method by performing combined FISH with morphological and immunohistochemical/immunofluorescence detection of cells (fluorescence immunophenotyping and interphase cytogenetics as a tool for the investigation of neoplasms; FICTION).

In this study, we examined cytogenetic abnormalities in newly diagnosed MM and compared the efficacy of three different enrichment modalities for FISH: direct FISH (n = 137), a FICTION technique (n = 224), and a plasma cell sorting FISH with FACS (n = 132).

## Results

### Baseline patient characteristics

**Figure 1:**
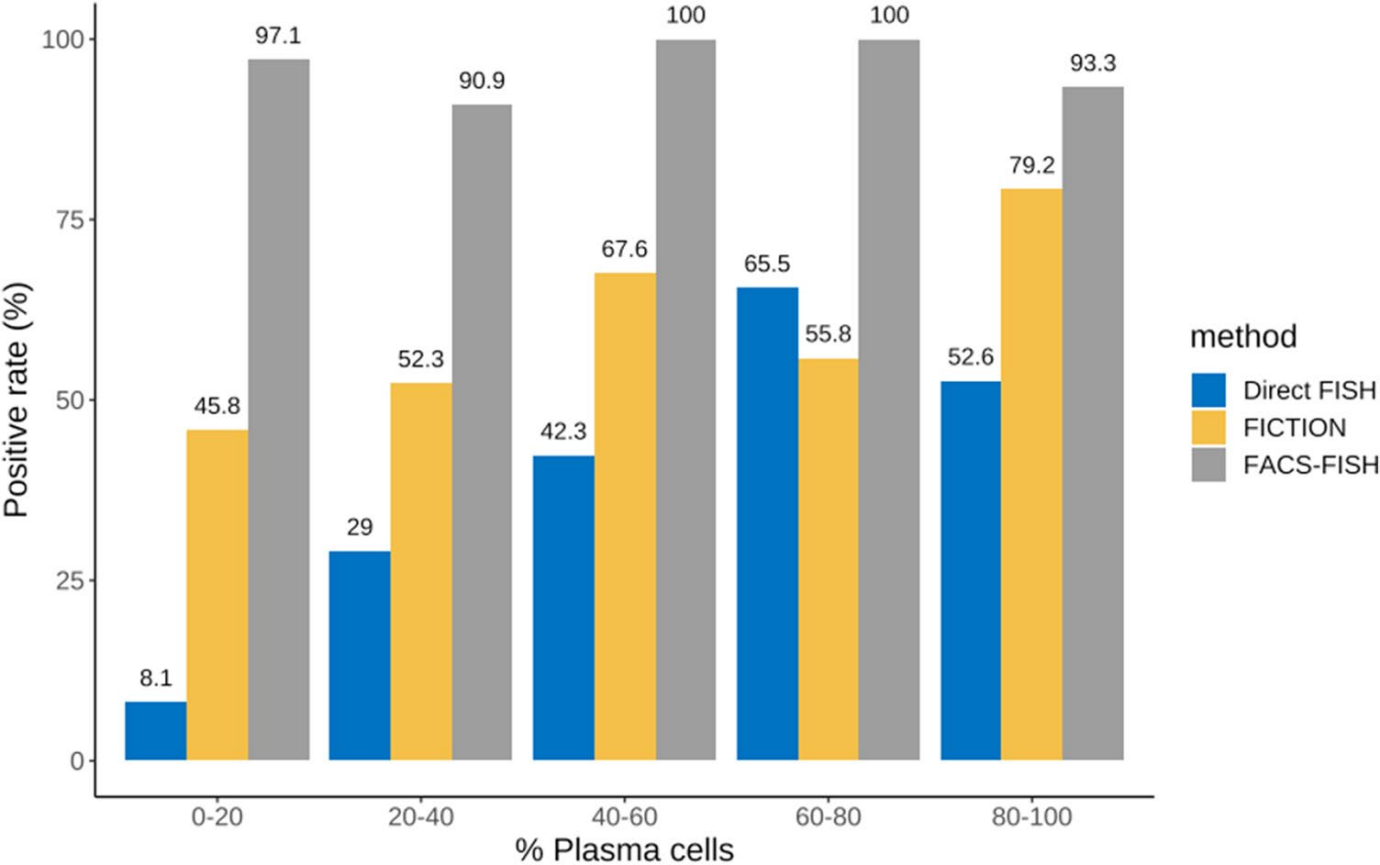
FISH positivity and plasma cell abundance according to the different enrichment methods in BM samples of MM patients. The positive rate of FISH differs according to the method and plasma cell abundance.

Among BM aspirates of 493 NDMM patients, 137 samples were tested by direct FISH, 224 samples were assessed by FICTION, and 132 samples were analyzed by FACS-FISH. There were no significant differences in baseline patient characteristics in terms of age, gender, isotype, serum creatinine, serum hemoglobin, serum calcium, percentage of bone marrow plasma cells, and international staging system (ISS) among these patients (Table 1). The positive rate of FISH differed according to the method (Fig. 1).

**Table 1:** Baseline characteristics of subjects in the three groups and *P* values for ANOVA.

Characteristics	Direct FISH (*n* = 137)	FICTION (*n* = 224)	FACS-FISH (*n* = 132)	*P* value
Age, median (range), years	64 (43–84)	65 (31–92)	67 (37–91)	0.061*
**Gender**				
Female	67 (48.9%)	112 (50.0%)	53 (40.2%)	0.780^†^
Male	70 (51.1%)	112 (50.0%)	79 (49.8%)	0.775^†^
**Immunoglobulin isotype**				
IgG	74 (54.0%)	113 (50.4%)	66 (50.0%)	0.756^†^
IgA	30 (21.9%)	40 (17.9%)	22 (16.7%)	0.467^†^
IgD	7 (5.1%)	10 (4.5%)	6 (4.6%)	0.890^†^
IgM	0 (0.0%)	1 (0.4%)	0 (0.0%)	0.588^†^
Light-chain only	26 (19.0%)	57 (25.4%)	37 (28.0%)	0.300^†^
Non-secretory	0 (0.0%)	3 (1.3%)	1 (0.8%)	0.245^†^
**Laboratory test, median (range)**				
Creatinine (mg/dL)	1.0 (0.46–16.45)	0.9 (0.43–19.57)	0.9 (0.5–10.1)	0.677*
Hemoglobin (g/dL)	11.1 (4.9–15.7)	11.5 (4.2–17.6)	11.9 (9.7–13.7)	0.113*
Calcium (mg/dL)	9.1 (7.4–13.7)	9.2 (7.5–17.1)	9.2 (7.3–15.1)	0.112*
Bone marrow plasma cell, median (range), %	43.5 (10.6–95.9)	35.3 (10.1–97.9)	35.2 (10.1–97.8)	0.100*
**ISS**				
I	23 (16.9%)	71(33.0%)	42 (37.8%)	0.059^†^
II	45 (33.1%)	74 (34.4%)	32 (28.8%)	0.082^†^
III	69 (50.7%)	73 (34.0%)	42 (37.8%)	0.028^†^
Missing	0	6	19	

Data are expressed as *n* (%) of patients, unless otherwise indicated.

*Kruskal–Wallis test.

^†^Chi-squared test.

ANOVA, analysis of variance; ISS, International Staging System for Multiple Myeloma.

### Detection rate of chromosomal abnormalities by FISH in NDMM patients

The overall detection rates among these three different methods were distinct (38.0% (52/137) by direct FISH vs 56.3% (126/224) by FICTION vs 95.5% (126/132) by FACS-FISH; *P* < 0.001) (Table 2). Deletion involving chromosome 17p was observed in 34.9% (24/224) by FACS-FISH, and this detection rate was significantly higher than that observed by direct FISH (5.8% (8/137), *P* < 0.0001) and FICTION (10.7% (24/224), *P* < 0.0001). Deletion involving chromosome 13q was observed in 52.3% (70/131) by FACS-FISH, and this detection rate was significantly higher than that observed by direct FISH (19.7% (27/137), *P* < 0.0001) and FICTION (22.8% (51/224), *P* < 0.0001). Translocation involving chromosome 14 was observed in 36.4% (48/132) by FACS-FISH, and this detection rate was significantly higher than that observed by direct FISH (17.7% (16/137), *P* < 0.0001) and FICTION (11.2% (25/224), *P* < 0.0001). Gains of 1q21 were observed in 46.2% (61/132) by FACS-FISH, and this detection rate was significantly higher than that observed by direct FISH (17.7% (11/62), *P* = 0.0007) and FICTION (23.4% (52/222), *P* < 0.0001).

**Table 2:** Positive rate of FISH according to enrichment method in NDMM patients.

		Direct FISH, % (*n*/total)		FICTION, % (*n*/total)		FACS-FISH, % (*n*/total)		*P* value
Deletion	17p13.1	5.8%	(8/137)^¶^	10.7%	(24/224)^¶^	34.9%	(46/132)^‡§^	< 0.0001*
	13q14.3	19.7%	(27/137)^¶^	22.8%	(51/224)^¶^	52.3%	(70/131)^‡§^	< 0.0001*
Translocation	t(11;14)	5.8%	(8/137)^§¶^	8.0%	(18/224)^‡¶^	24.2%	(32/132)^‡§^	< 0.0001*
	t(4;14)	5.8%	(8/137)	3.1%	(7/224)^¶^	9.9%	(13/132)^§^	0.0299*
	t(14;16)	0.0%	(0/137)	0.0%	(0/224)	2.3%	(3/132)	0.0188^†^
Gains	1q21	17.7%	(11/62)^¶^	23.4%	(52/222)^¶^	46.2%	(61/132)^‡§^	< 0.0001*
Overall positive rate		38.0%	(52/137)^‡§^	56.3%	(126/224) ^‡¶^	95.5%	(126/132) ^‡§^	< 0.0001*

*Chi-square test.

^†^Fisher-exact test.

^‡^Significant difference with direct FISH (Post hoc test).

^§^Significant difference with FICTION (Post hoc test).

^¶^Significant difference with FACS-FISH (Post hoc test).

NDMM, newly diagnosed multiple myeloma; FISH, fluorescence in situ hybridization; FICTION, fluorescence immunophenotyping and interphase cytogenetics as a tool for the investigation of neoplasms; FACS, fluorescence activated cell sorting.

### Plasma cell abundance and FISH detection rate in NDMM patients

**Figure 2:**
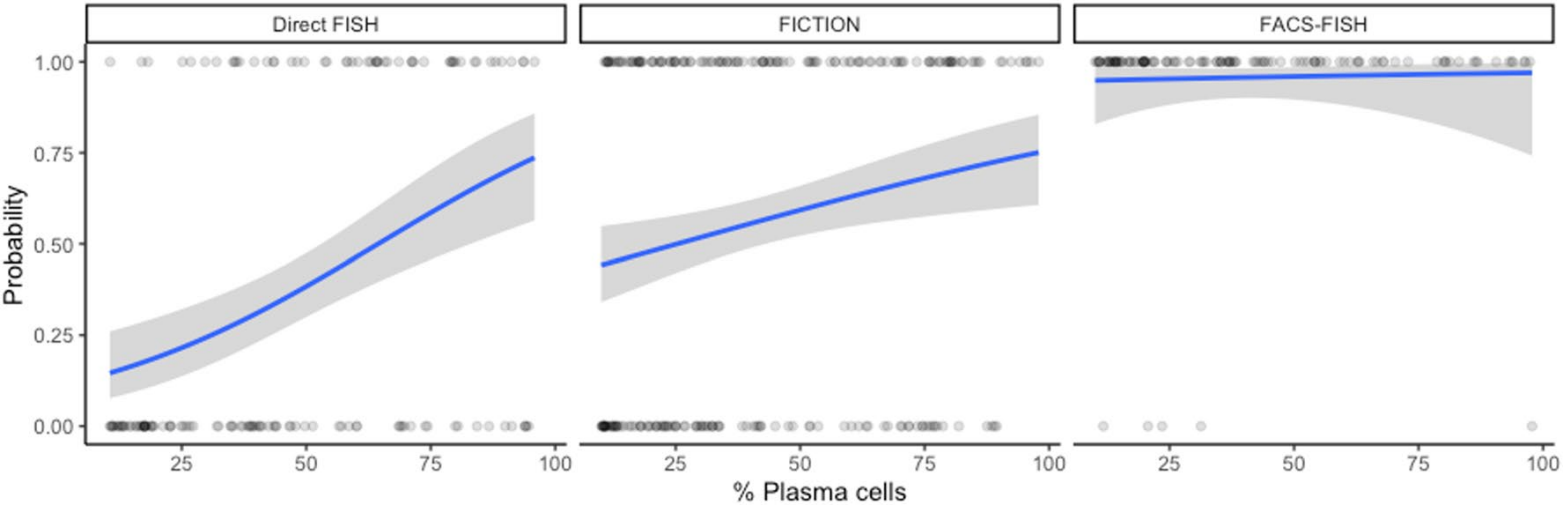
The effect of plasma cell percentage on FISH positivity. Logistic regression was performed to examine the effect of plasma cell percentage on FISH positivity according to the enrichment method. In direct-FISH, plasma cell percentage significantly influences the positivity of FISH with an OR of 1.04 for each 1% increase in plasma cell percentage (95% CI 1.01–1.08, *P* value = 0.006). In FICTION, plasma cell percentage significantly influences the positivity of FISH with an OR of 1.02 for each 1% increase in plasma cell percentage (95% CI 1.01–1.03, *P* value = 0.003). In FACS-FISH, the influence of plasma cell percentage on the positivity of FISH was not significant with an OR of 1.09 for each 1% increase in plasma cell percentage (95% CI 1.00–1.32, *P* value = 0.186).

To determine whether plasma cell frequency affects the detection rate of FISH and whether it differs by enrichment modalities, we performed logistic regression that examined the effect of plasma cell frequency on FISH positivity according to each method (Fig. 2). In direct FISH, plasma cell percentage significantly influenced FISH positivity with an odds ratio (OR) of 1.04 [95% confidence interval (CI): 1.01–1.08, *P* = 0.006] for each 1% increase in percent plasma cells. In FICTION, the percentage of plasma cells also significantly influenced FISH positivity with an OR of 1.02 (95% CI: 1.01–1.03, *P* = 0.003) for each 1% increase in percent plasma cells. However, plasma cell percentage and the positivity of FISH were not significantly associated in FACS-FISH (OR 1.09; 95% CI: 1.00–1.32, *P* = 0.186).

### Enumeration of cells with abnormal signals detected by FISH in NDMM patients

**Figure 3:**
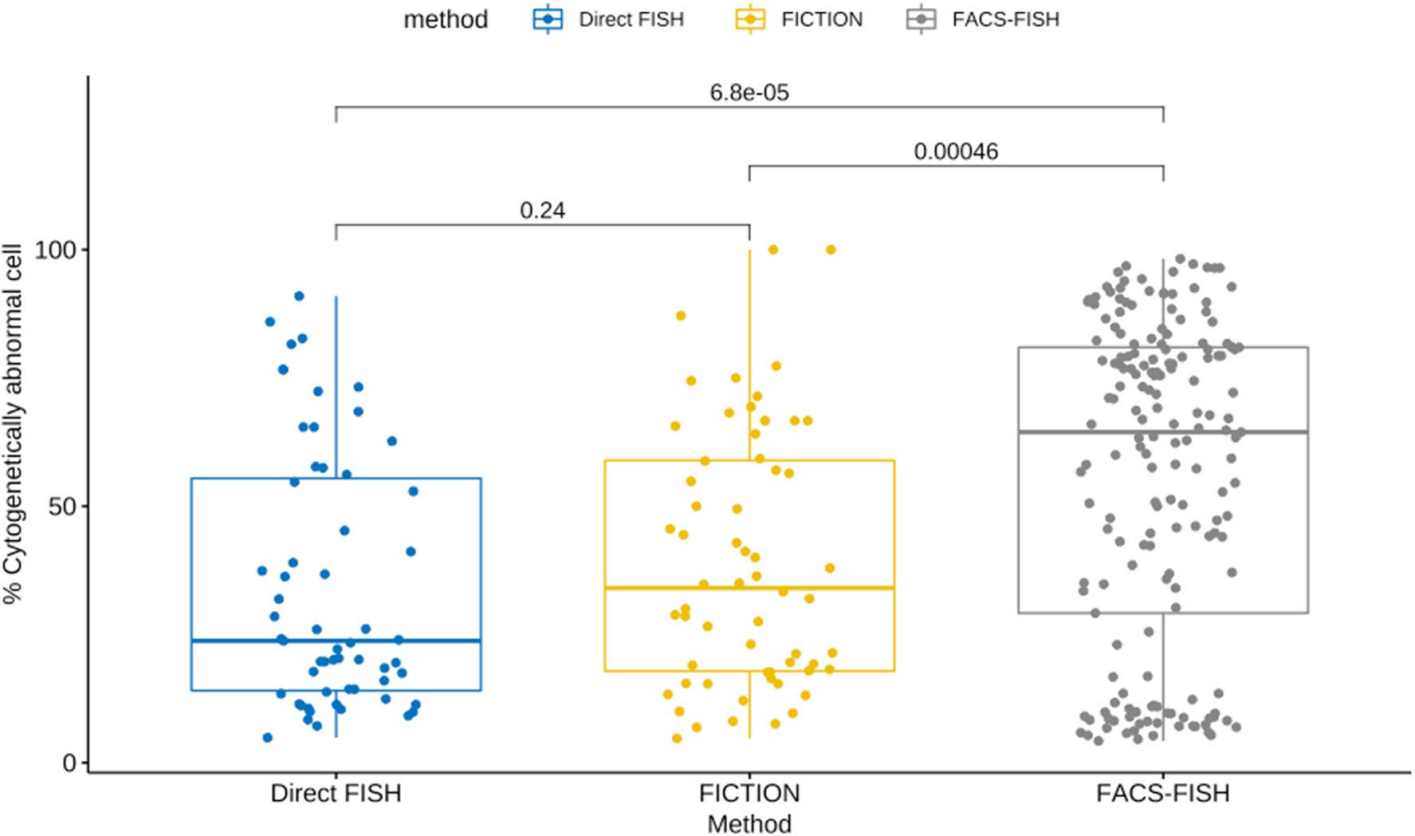
Boxplot showing percentage of cells with abnormal signals detected by FISH according to enrichment. When comparing the percentage of cells in which deletion 17p13.1, deletion 13q14.1, t(4;14), t(11;14), t(14;16) and gains of 1q21 were detected, FACS-FISH detected significantly higher numeric values of the abnormalities than the direct FISH and FICTION methods (Mann–Whitney-Wilcoxon test).

Next, we compared the percentage of cells with abnormal signals detected by FICTION and FACS-FISH according to the percentage of plasma cells in BM aspirates (Fig. 3). The median percentage of cytogenetically abnormal cells by FACS-FISH was 67.8%, which was significantly higher than 30.0% by FICTION (*P* < 0.001) (Fig. 3). The proportion of cases in which abnormal cells were detected in a low fraction of less than 10% was highest in order of FACS-FISH (18.1%), FICTION (10.0%), and direct FISH (9.8%).

**Figure 4:**
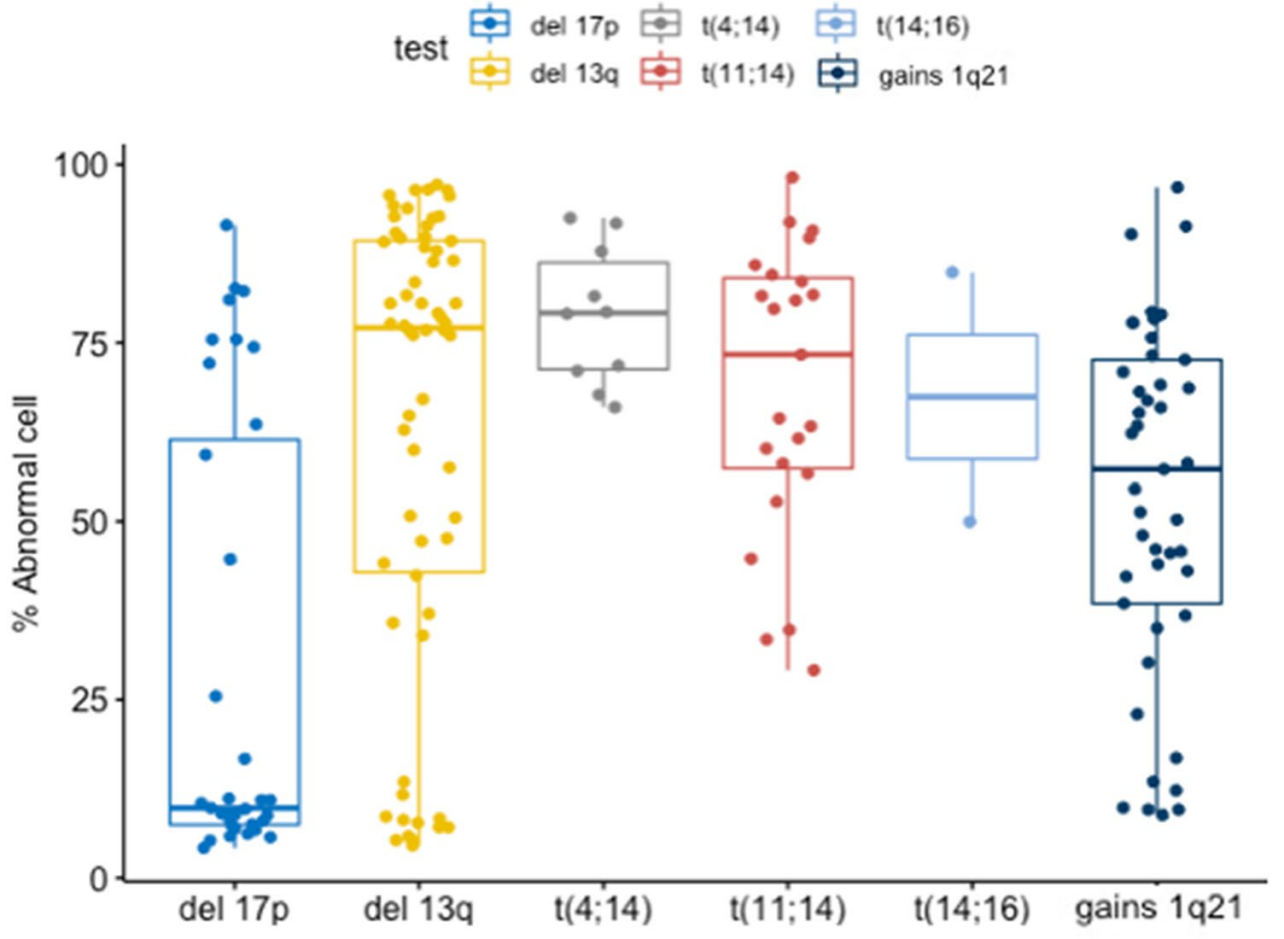
Boxplot showing percentages of detected abnormal cells by FACS-FISH according to cytogenetic abnormalities. Median percentage of detected abnormality of deletion 17p13.1, deletion 13q14.1, t(4;14), t(11;14), t(14;16) and gains of 1q21 were 9.8%, 77.1%, 79.2%,73.4%, 67.4% and 57.3% respectively.

Percentages of plasma cells with abnormal signals detected by FACS-FISH according to the type of cytogenetic abnormalities are presented in a box plot (Fig. 4). The median percentage of detected abnormal cells was lowest in the order of deletion 17p13.1, gains of 1q21, t(14;16), t(4;11), deletion 13q14.1, and t(11;14) (9.8%, 57.3%, 67.4%, 73.4%, 77.1%, and 79.2%, respectively).

## Discussion

In this study, we analyzed the efficacy of direct FISH, FICTION, and FACS-FISH in detecting cytogenetic abnormalities in BM samples from a large number of MM patients. We demonstrated the importance of FACS-FISH in improving the sensitivity of FISH for accurate detection of cytogenetic abnormalities. The overall detection rate of cytogenetic abnormalities by FISH reported in previous work differs depending on the number of probes and cells analyzed and the difference in analysis capability.^6,7^ For example, even if the same enrichment method is used, when 5 to 6 probes were used, the overall detection rate of cytogenetic abnormalities was 64.2 to 69.0%, and when 10 probes were used, the overall detection rate was reported as 96% using MACS. ^8–10^ However, the most important variable influencing FISH testing is indeed the enrichment modality.^11^ The FICTION method was described in 1992 by Weber-Mat-thiesen et al. as the first technique to combine immunophenotyping and FISH, enabling evaluation of immunophenotype and hybridization signals simultaneously under a fluorescence microscope.^12^ In the present study, chromosome abnormalities were identified in 56.3% (126/224) of MM patients using FICTION, similar to that reported (53%-69%) in previous studies.^13,14^ The detection rate of cytogenetic abnormalities with FICTION was significantly higher than direct FISH of 36.6% (52/142) shown in this study. However, FICTION requires an additional laborious cytomorphologic selection process of plasma cells, which are frequently interfered with by background signals overlapping target signals.^15^ In this regard, plasma cell enrichment processes such as MACS or FACS are employed to eliminate the need to manually identify plasma cells, resulting in higher throughput. Plasma cell targeting FISH using MACS was reported to show detection rates as high as 64.2% (120/187, 5 probes) to 69.0% (257/373, 6 probes), which is significantly higher than that of direct FISH.^8,9^ However, MACS can be unsuccessful if the percentage of plasma cells in the sample is very low or when plasma cells are dim or negative for the MM plasma cell surface marker CD138.^16,17^ In this work, we demonstrated that chromosomal abnormalities were identified in 95.5% (126/132) of cases of MM patients by FACS-FISH, consistent with the study finding more than one chromosomal abnormality in 96% of patients using FACS-FISH.^18^ Furthermore, we found that positivity of FISH by direct FISH and the FICTION method correlates with the percent plasma cells in BM cells of MM patients, while FISH results after enrichment with FACS were not significantly influenced by plasma cell abundance.

A limitation of this study is that the two methods could not be compared in parallel. It cannot be excluded that differences in the characteristics of the patient groups, especially differences in severity, or used FISH probes influence the comparison of positive rates. However, considering that the severity of the patients was rather high during the direct FISH test period with a low positivity rate, the difference cannot be due to the difference in patient groups. In the case of FISH probes, the internal verifications of analytical performance were conducted whenever the manufacturer or lot was changed, so it was judged that the impact on the results would not be significant.

Taken together, we show that the FISH results are highly dependent on the enrichment method and demonstrate that FACS enables improved FISH sensitivity regardless of the plasma cell abundance in BM samples from MM patients. Our work supports target enrichment using FACS to minimize false-negative results of FISH, especially in MM patients with a low tumor burden.

## Methods

### Patients and samples

This study was approved by the Institutional Review Board of Severance Hospital, Yonsei University College of Medicine, Seoul, Republic of Korea (IRB No. 4–2020-1383). Informed consent was waived for this retrospective study that evaluated anonymized samples and data and involved no potential risk to patients. We obtained BM aspirates from 493 newly diagnosed MM (NDMM) patients from February 2010 to March 2020 (Table 1). The percentage of bone marrow plasma cells were enumerated from 400-cell differential count on bone marrow aspirate smears. The ISS stages were defined as the 2005 International Myeloma Working Group (IMWG) recommendations.^19^ Since the subject of our study is about tools that detect genetic abnormalities, we used the ISS instead of R-ISS^20^ to eliminate the influence of the technique on the patient's stage. For cytogenetic analysis, BM aspirates were collected into sodium heparin-coated tubes (BD Biosciences, San Jose, CA). All methods were performed in accordance with the relevant guidelines and regulations.

### Fluorescent in situ hybridization (FISH)

For direct FISH, cell pellets were isolated by centrifugation of BM aspirates at 200 g for 10 min. The cell pellets were then incubated at 37 ℃ in 0.068 M potassium chloride for 30 min and fixed with 3:1 methanol/acetic acid. Fixed cells were dropped onto microscope slides and air-dried. Denaturation of target DNA and probe at 75 °C for 5 min and hybridization at 37 °C for 17 h were performed with a Thermobrite® Slide Processing System (Abbott Molecular, Abbott Park, IL, USA). After post hybridization wash steps with phosphate-buffered saline (PBS), nuclei were counterstained with 4',6-diamidino-2-phenylindole (DAPI).

The MM FISH panel consisted of six sets of commercial probes from two manufacturers (Metasystems GmbH, Altlussheim, Germany or Abbott Molecular, IL, USA); XL *TP53*/17cen dual spot or Vysis LSI *TP53*/CEP 17 FISH Probe Kit, XL *DLEU*/*LAMP* dual spot or Vysis LSI 13 *RB1* (13q14) SpectrumOrange Probe, XL t(4;14) *FGFR3*/*IGH* dual fusion or Vysis LSI IGH/FGFR3 Dual Color Dual Fusion Probes, XL t(11;14) *MYEOV*/*IGH* dual fusion or Vysis LSI *IGH/CCND1* XT Dual Color Dual Fusion Probes, XL t(14;16) *IGH*/*MAF* dual fusion or Vysis LSI *IGH/MAF* DF FISH Probe Kit, and XL *CDKN2C*(1p32) /*CKS1B*(1q21) dual spot probes. For each set of probes, two hundred interphase nuclei were analyzed. The positive cut-off level was set at 10% for fusion or break-apart probes and 20% for numerical abnormalities according to the recommendation by the European Myeloma Network^5^.

### Fluorescence immunophenotyping and interphase cytogenetics as a tool for the investigation of neoplasms (FICTION)

After post hybridization wash steps, slides were washed with PBS and air-dried. Fluorescein isothiocyanate (FITC)-conjugated polyclonal rabbit antibodies against human kappa and lambda light chains were applied to stain plasma cells. After incubation and washing, slides were counterstained with DAPI, and 200 plasma cells were assessed for each analysis.

### Fluorescence activated cell sorter (FACS)-FISH

About 3 mL of BM aspirate collected in a sodium heparin tube was centrifuged at 2100 rpm for 5 min. Then about 2 mL of buffy coat was isolated from BM aspirates was incubated with erythrocyte lysis buffer. After incubation at room temperature for 20 min, samples were centrifuged at 2100 rpm for 5 min. The cell pellet was then washed three times with PBS, and the cell concentration was adjusted to 1 × 10^6^–4 × 10^7^ cells/mL. Antibody staining was performed using anti-CD38-FITC and anti-CD138-PE (Beckman Coulter, CA, USA), and plasma cells were sorted with the BD FACSMelody™ (BD Biosciences, San Jose, CA, USA) or S3e™ Cell Sorter (Bio‐Rad Laboratories, Hercules, CA, USA). Cell gating and acquisition was done with FlowJo™ (BD Biosciences) or ProSoft™ softwares (Bio‐Rad Laboratories). To achieve high purity (target: ≥ 90% of sorting efficiency), the event rate was adjusted to 1,200 to 1,700 cells per second. The median of sorted events in samples of NDMM patients was 62,146 (range, 12,007–270,962). The median sorting efficiency was 90% (range, 84%–100%). Cytospin preparations were prepared by centrifugation of 220 µL of cell suspension at 800 rpm for 4 min in an Epredia™ Cytospin™ 4 Cytocentrifuge (Thermo Fisher Scientific, Waltham, MA, USA).

### Statistical analysis

The Shapiro–Wilk test was conducted to assess whether continuous variables followed the normal distribution. Age, creatinine, hemoglobin, calcium, and the percentage of bone marrow plasma cells among total nucleated cells were not normally distributed. The Kruskal–Wallis test and Mann–Whitney-Wilcoxon test were performed among the three groups or two groups with different methods, respectively. Categorical variables were expressed as percentages and were compared by the chi-square test. Post hoc Tukey's test was used to find the pairwise significance between groups. Logistic regression was performed to investigate the association of plasma cell percentage with FISH positivity. A two-sided *P* of less than 0.05 was considered statistically significant. Statistical analyses were performed with R version 4.0.0 (R Foundation for Statistical Computing, Vienna, Austria).

## Supplementary Information

Supplementary Information.
