# Supplementary material for: Cytogenetic testing by fluorescence in situ hybridization is improved by plasma cell sorting in multiple myeloma
Source: Sci Rep. 2022 May 18;12:8287. doi: 10.1038/s41598-022-11676-w (PMC9117238; doi:10.1038/s41598-022-11676-w)
Supplement: Supplementary file 1 — Supplementary Information. [file 41598_2022_11676_MOESM1_ESM.docx]

| Plasma cell (%) | Direct FISH | | FICTION | | FACS-FISH | |
| --- | --- | --- | --- | --- | --- | --- |
| 0-25% | 27.0% | (37/137) | 35.7% | (51/132) | 38.6% | (80/224) |
| 25-50% | 31.4% | (43/137) | 27.7% | (39/132) | 29.5% | (62/224) |
| 50-75% | 23.4% | (32/137) | 19.2% | (25/132) | 18.9% | (43/224) |
| 75-100% | 18.2% | (25/137) | 17.4% | (17/132) | 12.9% | (39/224) |

Supplementary Table S1. Distribution of percentages of plasma cell infiltration on bone marrow aspirate smear according to each method

Supplementary Table S2. Distribution of maximum frequency of aberrations detected according to each method

| Maximum frequency of aberrations detected (%) | Direct FISH | | FICTION | | FACS-FISH | |
| --- | --- | --- | --- | --- | --- | --- |
| 0-25% | 16.8% | (23/137) | 19.2% | (43/224) | 13.6% | (18/132) |
| 25-50% | 8.0% | (11/137) | 17.9% | (40/224) | 6.8% | (9/132) |
| 50-75% | 7.3% | (10/137) | 11.6% | (26/224) | 21.2% | (28/132) |
| 75-100% | 5.1% | (7/137) | 7.6% | (17/224) | 53.8% | (71/132) |

Supplementary Table S3. Distribution of purity of available samples (n=80) enriched by FACS.

| Purity (%) | FACS-FISH | |
| --- | --- | --- |
| 75-80% | 1.3% | (1/80) |
| 80-85% | 3.8% | (3/80) |
| 85-90% | 26.3% | (21/80) |
| 90-95% | 65% | (52/80) |
| 95-100% | 3.8% | (3/80) |
